# A reassessment of the role of joint receptors in human position sense

**DOI:** 10.1007/s00221-023-06582-0

**Published:** 2023-03-03

**Authors:** Uwe Proske

**Affiliations:** School of Biomedical Sciences, Monash University, PO Box 13F, Clayton, VIC 3800 Australia

**Keywords:** Muscle spindle, Thixotropy, Proprioception, Position sense, Joint receptor

## Abstract

In the past, the peripheral sense organs responsible for generating human position sense were thought to be the slowly adapting receptors in joints. More recently, our views have changed and the principal position sensor is now believed to be the muscle spindle. Joint receptors have been relegated to the lesser role of acting as limit detectors when movements approach the anatomical limit of a joint. In a recent experiment concerned with position sense at the elbow joint, measured in a pointing task over a range of forearm angles, we have observed falls in position errors as the forearm was moved closer to the limit of extension. We considered the possibility that as the arm approached full extension, a population of joint receptors became engaged and that they were responsible for the changes in position errors. Muscle vibration selectively engages signals of muscle spindles. Vibration of elbow muscles undergoing stretch has been reported to lead to perception of elbow angles beyond the anatomical limit of the joint. The result suggests that spindles, by themselves, cannot signal the limit of joint movement. We hypothesise that over the portion of the elbow angle range where joint receptors become active, their signals are combined with those of spindles to produce a composite that contains joint limit information. As the arm is extended, the growing influence of the joint receptor signal is evidenced by the fall in position errors.

## Introduction

When my forearm is in the middle of its movement range and it is not moving, I have no conscious awareness of any sensation arising from it. Yet if I close my eyes, I can envisage its position and if this is tested in an experiment, my imagined position is quite accurate (Chen et al. 2021). Then, if I move the arm sufficiently far into extension, apart from experiencing the movement sensation, I now begin to perceive sensations coming from the static arm. I can feel stretch of skin over the inside of the forearm and a slightly altered sensation coming from the joint. Such everyday experiences are relevant to a discussion of the sensory receptor origin of position sense and the three candidates for its generation: muscle receptors, skin receptors and joint receptors.

Up until the middle of the twentieth century, it was believed that joint receptors were the principal position sensors. However, during subsequent years strong experimental evidence emerged which supported the view that muscle spindles were responsible for the position signal over most of the range of joint angles. For a detailed account of these changing views, see McCloskey (1978). With the acceptance of spindles as position sensors, joint receptors were assigned a relatively minor role, that of joint limit detectors. That is, as the joint was rotated towards its anatomical limit, joint receptors were thought responsible for signalling the approaching limit (McCloskey 1978; Rossi and Grigg 1982; Fuentes and Bastian 2010). For more recent reviews of joint receptors and their role in proprioception, see Gilman (2002), Macefield (2005) and Macefield (2021).

In the present account, we consider that perhaps we have dismissed too lightly a possible role for joint receptors in kinaesthesia. Here, we are reminded of the words by Peter Matthews (1972, p 492), “The repeated experimental testing of traditional beliefs appears to be an unfortunate necessity of life.”

Joint receptors have an “activation angle” (Burgess and Clark 1969). It is the angle of the joint, somewhere near the limit of its movement range, where a slowly adapting joint afferent begins to discharge tonically. Depending on their location within the joint capsule, slowly adapting joint receptors have different activation angles (Rossi and Grigg 1982). When the joint is rotated towards the limit of its movement range, proximity to the limit is signalled by the number of joint receptors activated and the rates of their discharge. Here, we propose that the muscle length signal provided by muscle spindles, and which does not contain any joint limit information, is combined with a joint afferent signal that is able to define the limit. In other words, while in the mid-range of joint angles the spindle signal is exclusively responsible for generating a position signal, only by combining it with afferent signals from the joint will a signal be generated that covers the full working range of the joint.

We have recently made some observations on the human forearm which suggest a contributory role for joint receptors in position sense. This was not just an action at the anatomical limit of movement but over a portion of the angular range before reaching that limit. We employed a relatively simple test to measure position sense, where the subject indicates the perceived position of their forearm, hidden from view, by pointing to where they think it is. The experimental arrangement involved strapping one of the subject’s forearms to a lightweight paddle. The paddle was provided with potentiometers at its hinge joint which generated a continuous signal of elbow angle. The arm and paddle were hidden behind a screen. The subject’s other arm was used to rotate a lever that moved a pointer paddle with which they indicated the perceived position of the hidden arm. For details, see Chen et al. (2021, Fig. 1B).

**Fig. 1:**
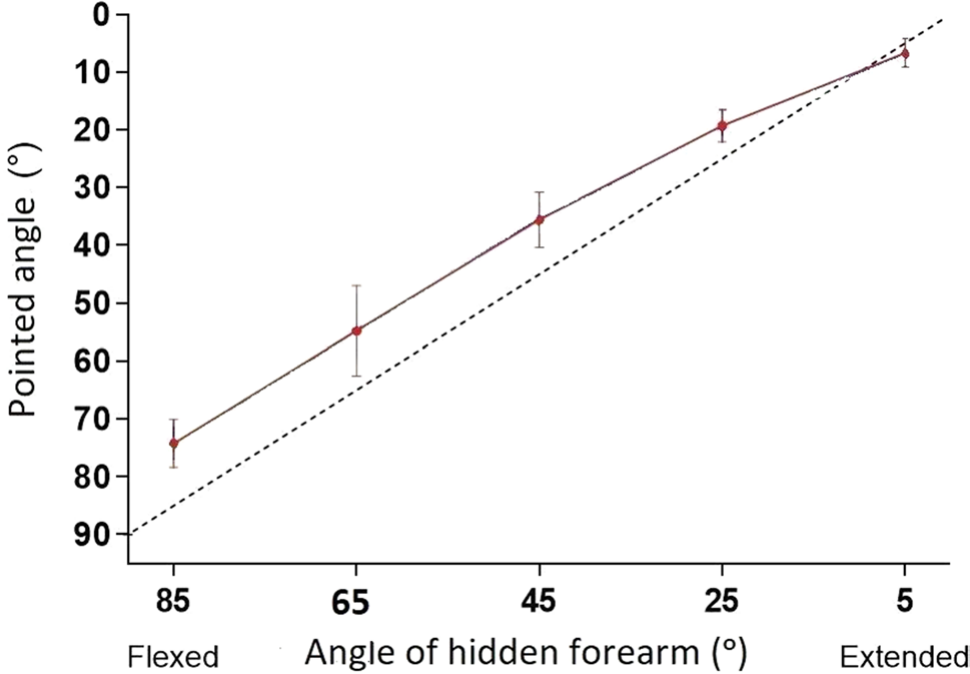
One-arm pointing task. Perceived position of a subject’s hidden forearm, indicated with a pointer, moved by the subject with their other arm. Abscissa, angle of the hidden forearm, ordinate, perceived angle indicated with the pointer. 0° is the angle of the fully extended forearm, 90° the angle when the position of the forearm was vertical to the supporting base. At each test angle, before making a measurement, the hidden arm was conditioned with isometric contractions of elbow flexors and extensors. At the test angles, 5°, 25°, 45°, 65° and 85°, presented in random order, are shown mean position errors for three repeated measurements for each subject, pooled for 10 subjects (means ± SD), representing a total of 30 measurements at each test angle. Solid line, line joining data points to indicate trend of the data. Dashed line, line of equality, the location of the hidden arm if its position had been determined accurately by the pointer. All values, except that for 5° lay above the line of equality, representing position errors in the direction of forearm extension. Redrawn from Chen et al. (2021)

Position sense was measured in the vertical (sagittal) plane over a range of angles, between 0° where the forearm was fully extended and 90° where it was at right angles to the supporting base. Unfortunately, the apparatus did not allow the exploration of angles in the direction of elbow flexion beyond 90°. At the start of a trial, one of the five randomly selected test angles was chosen (5°, 25°, 45°, 65° or 85°) and the experimenter moved the relaxed arm, hidden from view, to that angle and locked it in position. The subject then carried out brief, voluntary, isometric contractions of elbow flexors and extensors to put them into a defined thixotropic state (Proske et al. 2014). Once arm muscles had relaxed, the arm was unlocked and the subject was asked to hold its position, while, with their other arm, they moved the pointer paddle to where it was perceived as aligned with the position of the hidden arm (Chen et al. (2021).

Figure 1 shows the mean pointing errors (± SD), at the five different test angles, for 10 subjects. It can be seen that for all of the measured angles, except 5°, values lay above the line of equality. Errors above the line, giving a lower value for the pointed angle, indicate a more extended forearm. That is, the subject believed their hidden arm to be more extended by about 10° than was actually the case. We had seen such trends in previous experiments on pointing (Tsay et al. 2016).

While we remain uncertain about the origin of this 10° offset, we considered the possibility that the spindle afferent signal coming from elbow flexors was larger than that coming from the extensors. If so, this would bias forearm position in the direction of elbow extension. Such an explanation is supported by reports of differences in the sizes of position errors generated by muscle vibration. Errors during vibration of flexors were several times larger than during vibration of extensors (Craske 1977; Lackner and DiZio 1992).

The object of the present account is to point out that as the arm was moved from its most flexed position (85°, extension error of 10.4° ± 4.1°, SD) into extension, the offset error into extension became progressively less and at 5° the position indicated lay slightly below the line of equality (flexion error of 1.9° ± 2.4°, SD). Why did position errors at the more extended angles lie closer to zero? There are three possible explanations. They are due to (1) thixotropy: a length-dependent change in influence of the conditioning contractions on pointing errors; (2) a progressive increase in influence of skin stretch receptors on position sense, as the arm is extended and skin over the inside of the elbow joint is stretched and (3) a growing influence from rising joint receptor activity signalling the approaching limit of movement at the joint.

Before discussing these possibilities further, it is necessary to emphasise that in the experiments described here position sense was measured in a one-arm pointing task. Unfortunately, there is no consensus over the best method to use in measuring position sense. For the forearm, there are three classes of measurement; two-arm position matching, one-arm pointing and one-arm position reproduction (Proske and Chen 2021). Position reproduction measurements have a large memory component. In two position reproduction studies of position sense measured at the shoulder joint it has been reported that towards the limit of joint movement there may, or may not, be an increase in position sense accuracy (Janwantanakul et al. 2001; Suprak 2011). It will be the aim of future experiments to resolve this uncertainty.

## Thixotropy

In a study of position sense, it is first necessary to contract the muscles acting at the joint being studied to put their spindles into a defined thixotropic state. A feature of spindles that have not been deliberately conditioned by a contraction is that some slack may be present in their intrafusal fibres as a result of a previous shortening movement. Such slack will lower the maintained rate of discharge in spindles and, therefore, alter the measured value of position sense. Removal of slack by the contraction leads to a rise in spindle resting activity, previously called, “post-contraction sensory discharge” (Hutton et al. 1973). In our position sense study at the elbow, we considered the possibility that the decline in position errors in the extended forearm was, in some way, related to a change in the thixotropic state of forearm muscles.

In the present study, at each test angle, before making position sense measurements, flexors and extensors had been conditioned by a contraction, removing any pre-existing slack in both groups. Therefore, when the forearm was moved to an extended test angle, the elbow flexors would be stretched by the movement and their spindles would generate high levels of discharge. At the same time, the extensors would be shortened, but any developing slack in them would be removed by the conditioning contraction. Nevertheless, since they were now being held at a shorter length than in the mid-range of muscle lengths, the extensors might be expected to have a lower maintained rate of afferent discharge because of the intrafusal length-tension relation of their spindles. The combination of a growing flexor signal and a declining extensor signal is unable to account for the falls in position errors at extended elbow angles.

In the plot of Fig. 1, we have shown, for simplicity, only the position error distribution after conditioning contractions of both antagonists. In the original experiments, two additional conditioning methods were used. In one, only flexors were conditioned, in the other slack was deliberately introduced in both antagonists. For details, see Chen et al. (2021). In the event, the error distributions for the three methods were quite similar. All three showed the decline in errors as the arm reached near full extension (Chen et al. 2021, Fig. 5). We concluded that the phenomenon of a reduction of position errors, as the limit of movement at the joint was approached, was not sensitive to differences in the thixotropic state of elbow muscles.

## Skin receptors

In recent years, there has been a trend to emphasise the role of skin receptors in kinaesthesia. Here, an important point is that, while there is good evidence for skin receptors participating in movement sense (Collins and Prochazka 1996; Collins et al. 2005), evidence for their contribution to position sense is less strong (Edin 2001). Since our experiments were concerned with position sense, we posed the question, what kinds of skin receptors might be involved in signalling position. The most likely candidates are skin stretch receptors of the slowly adapting Type II kind, (Proske and Gandevia 2012, p 1660). They would be expected to respond as skin over a joint was stretched during flexion or extension movements.

There is some more recent information on the possible role of skin receptors in position sense. In human subjects with a sensory and autonomic neuropathy (HSAN III), limb muscles are believed to lack muscle spindles. In a two-leg matching task, position sense at the knee was poor, but improved after applying kinesiology tape to both knees. It was postulated that the tape increased the kinaesthetic signal of cutaneous origin and the findings suggested a partial take-over of the role of spindles by skin receptors (Macefield et al. 2016). At the forearm, there was no difference in position sense accuracy between HSAN III and normal subjects and taping of the joint did not improve performance. Here, it was postulated that skin receptors had fully taken over the role of muscle spindles as position sensors (Smith et al. 2020).

Care should be taken in extrapolating from observations on subjects suffering from a severe peripheral neuropathy. However, it does seem that under certain conditions skin receptors can replace spindles as position sensors. We would add that to generate a fully functional position signal, the skin signals would have to combine with signals from joint receptors to acquire the necessary joint limit information.

## Joint receptors

Quite a lot is known about joint receptors, based largely on animal experiments carried out during the 1950s to 1980s. Given that this discussion is about position sense, the only suitable joint receptors implicated are those with slowly adapting properties. A number of detailed observations have been made using the cat knee joint as a model. Here care must be taken in identifying true joint afferents. Occasional afferents of muscle spindles from the nearby popliteus muscle may take an aberrant course, travelling via the joint nerve (McIntyre et al. 1978).

Joint receptors with a slowly adapting discharge are the Ruffini endings lying within the joint capsule. The stimulus for the receptors is stretch of the capsule, rather than transverse compression (Grigg and Hoffman 1982). Only at extreme angles is the capsule stretched. Stretch or contraction of adjacent muscles can act to stretch the capsule. For the primate knee, force from stretch or contraction of gastrocnemius was effective in activating joint receptors, but only if the knee was already in an extended position (Grigg and Greenspan 1977).

These observations suggest that any forces acting to stretch the joint capsule, be they an extension torque exerted by the weight of the arm, or stretch or contraction of muscles acting at the joint, are likely to facilitate responses of joint receptors. Loading the arm by asking the subject to support a weight should have similar effects.

The data on signalling properties of joint receptors, on which the present account is based, come from Burgess and Clark (1969) and Ferrell (1980) for the knee joint of the cat and Grigg and Greenspan (1977) for the primate knee joint. The general impression is that the majority of joint receptors are signalling at or near full extension of the joint. A flexion response often requires strong flexion combined with adduction/abduction at the joint. Under these conditions, most extension units will also respond to flexion. Flexion–extension units represent by far the largest group of joint receptors (40–70%) identified in an experimental sample. There were many fewer extension-only or flexion-only units. The summary impression is that the sensory innervation in the cat and primate knee joint favours signalling extension at the joint, although strong flexion will initiate some, if less powerful, responses. It is not clear why there is such a non-symmetrical signalling capacity at the two extremes of joint movement. It occurred to us that the fully extended human elbow joint may be more susceptible to damage from over-extension, as the weight of the arm bears down on it, compared with a fully flexed joint, where the joint is somewhat protected by the presence of the upper arm against which the forearm folds.

In their account, Burgess and Clark (1969) introduced the term, “activation angle”. This was the angle at which a joint receptor would begin to discharge as the limb was rotated towards the limit of the movement range. For flexion/extension units the activation angle might be 15°, that is, the unit would commence discharging at 165° and increase its discharge rate until it peaked at 180° (the anatomical limit for extension). Other reports were for activation angles of 30° (Grigg and Greenspan 1977) and 20° (estimated from Fig. 2, Ferrell 1980). Here the point is that signalling by joint receptors is not restricted to the anatomical limit of the movement range, but is often initiated at angles short of the limit.

While a joint receptor may peak in its discharge at the anatomical limit, if the joint is forced beyond the limit, without causing irreversible damage, the discharge may continue at its peak, (Burke et al. 1988) or fall (Grigg 1975; Clark et al. 1979). That is, it is a feature of joint receptor responses that their discharge peaks when the point of damage is approached, unlike the spindle signal which does not appear contain any information about the approaching limit (Craske 1977). To conclude, slowly adapting joint receptors would begin responding well short of the anatomical limit of the joint. As the limit was approached, more units would be recruited and those already active would increase their discharge rate further. The peak discharge of the population would be reached at the anatomical limit, and beyond the limit no further increases would be anticipated.

In Fig. 1 it can be seen that the offset error, in the direction of extension, began to fall at about 25° short of the extension limit. With further extension, there was a steady decline in error until it had reached close to zero. That is, as the arm was extended, its perceived and actual positions began to more nearly coincide. We hypothesise that as the arm was extended, the rising spindle signal coming from elbow flexor muscles was joined by a growing signal from joint receptors sensitive to joint extension. This combined signal would provide more accurate information about forearm position than a spindle or joint receptor signal on their own. The same argument would apply to movements into flexion; here the extensor spindle signal would combine with responses from flexion-sensitive joint receptors.

There have been reports of experiments on human subjects where position sense was measured at the knee joint after anaesthetising skin adjacent to the knee and infiltrating the knee joint capsule with anaesthetic (Clark et al. 1979). It was found that anaesthesia did not affect subjects’ position sense. However, this study, like a number of others, restricted observations to the mid-range of knee angles where joint receptors were not likely to be active. It was therefore concerned only with mid-range responses and did not address the question of a role for joint receptors under conditions where they were likely to contribute.

## Discussion

It has been postulated that the discharge rate: muscle length relation for muscle spindles, used to determine the length of the muscle (angle of the joint), has been calibrated by the brain during development; as the developing young watches their arm moving, this relation is established (Held and Bower 1967). Vibration of a muscle selectively engages its muscle spindles (Goodwin et al. 1972). Craske (1977) reported that vibration of a lengthening muscle can generate illusions of changes in limb position that exceeded the anatomical limit of the joint (see also Lackner and DiZio 1992). Craske proposed that, in an attempt to account for the unphysiologically high discharge rate of spindles evoked by vibration, the brain extrapolated, using the calibrated spindle discharge rates occurring over the normal working range of the joint, to generate perception of anatomically impossible angles. Here the important conclusion is that during limb movement there appears to be no component of spindle discharge that is able to indicate the approaching anatomical limit of the joint. We propose that the role of joint receptors is to signal that limit.

Our working hypothesis is that as the arm was moving through its normal working range, its position would be determined by the brain, based on the established spindle discharge rate: muscle length relation. This information would then be forwarded to a central map, indicating position of the arm, relative to the rest of the body (see, for example, Matthews 1972). If the arm was sufficiently close to its anatomical limit of movement, some activity would be generated in joint receptors as well. This, too, would be assessed centrally; here, the joint afferent information would be interpreted in terms of the position of the arm relative to the limit of joint movement. The central representation of the joint afferent signals would therefore be distinct from that for spindles, since the spindle-based representation would not contain any joint limit information. At some point in the brain, presumably further upstream, the spindle and joint signals would be combined to construct a signal that represented limb position more accurately, as shown by the declining position errors as the arm was fully extended (Fig. 1).

In one experiment, Craske (1977) asked subjects to describe their sensory experiences when they attempted to move their arm to voluntarily overcome the reflex contraction (tonic vibration reflex) of their biceps muscle in response to its vibration. While the majority of subjects had difficulty in moving their arm, significantly, four subjects reported experiencing double or multiple images of their forearm. In commenting on this observation, Craske (1977) concluded that for these subjects muscle and joint information were not perfectly integrated and that each gave rise to a separate sensory experience. Such an observation supports the proposal for a separate central representation of joint and muscle signals. If we are correct, our observation on the length dependence of position errors is the result of a combination of these two sources of information to generate the final position signal.

## Conclusions

In the future, we plan to modify our experimental arrangement to allow measurement of arm position over the full working range of the forearm, to include angles where the arm is fully flexed. Our prediction is that for the flexed elbow there may be reductions in position errors similar to those seen at full extension, although the effects are likely to be less pronounced. The reason is that the animal data indicates that the majority of joint receptors favour signalling joint extension rather than flexion (see P7).

Concerning the question of the combination, centrally, of joint and spindle signals, it is interesting to go back to earlier literature. Poggio and Mountcastle (1963) found 26% of third-order cells in the ventrobasal complex of the thalamus were sensitive to joint movements in the contralateral limb. Each neurone responded to rotation of the joint in one direction only. Its discharges reached their maximum at either full flexion or full extension and never at any intermediate position. The range of excitatory angles of these neurones was wide, about four times as wide as had been reported for joint receptors (Mountcastle et al. 1963). The observations of neurones exhibiting tonic activity, with a wide excitatory range, and a clear peak in their discharge at the joint limit, suggest that at the level of the thalamus signals from joint and spindle receptors may have already been combined.

Poggio and Mountcastle (1963) commented on how secure transmission was across synapses in the three-neurone pathway between putative joint receptors and the thalamus. This observation is consistent with findings using microstimulation (Macefield et al. (1990). Stimulation of single joint afferents evoked conscious sensations. By comparison, stimulation of single muscle afferents had no effect and it was necessary to stimulate a population of afferents to evoke any perceptual effects (Gandevia 1985). Interestingly, in the Macefield study, stimulating single skin afferents also evoked conscious sensations, but not stimulation of cutaneous Type II receptors signalling skin stretch.

Secure central transmission of joint receptor signals presumably reflects the importance of the information in providing protection against damage to the joint. An example of such a protective influence has recently been provided by Porssut et al. (2022) in a virtual reality study. If the user felt that their own arm was near its anatomical limit into extension, they rejected the avatar as their body, no matter what posture the avatar’s arm had adopted. If the user’s arm was held short of full extension, all postures of the avatar’s arm were accepted, including when it appeared to be at the limit of its extension. These findings may have broader implications for embodiment processes. For example, in the rubber hand illusion (Botvinick and Cohen 1998), embodiment of the rubber hand may well be influenced by the position of the hidden, real hand if this adopts a posture close to the limit of its movement.

Does any of this matter? Osteoarthritic joint disease presents frequently in the clinic. We estimate that the influence of joint receptors on spindle-based position sense can occur over as much as 30% of the total movement range at the elbow joint. Joint disease can impair position sense (McDougall 2019); even a partial loss of position sense is a serious, debilitating condition (Cole 2016). Appreciating the role played by joint receptors in position sense helps us better understand the source of that debilitation.

## Data Availability

Data sharing is not applicable to this article as no datasets were generated or analysed during the current study. The data cited is all contained in the article by Chen et al. (2021), Exp Brain Res 239: 675–686 https://doi.org/10.1007/s00221-020-05999-1.
